# Autophagy protects against dasatinib-induced hepatotoxicity via p38 signaling

**DOI:** 10.18632/oncotarget.3357

**Published:** 2015-01-31

**Authors:** Xiaochun Yang, Jincheng Wang, Jiabin Dai, Jinjin Shao, Jian Ma, Chao Chen, Shenglin Ma, Qiaojun He, Peihua Luo, Bo Yang

**Affiliations:** ^1^ Institute of Pharmacology & Toxicology, College of Pharmaceutical Sciences, Zhejiang University, Hangzhou, China; ^2^ Center for Drug Safety Evaluation and Research of Zhejiang University, Hangzhou, China; ^3^ Nanjing Medical University, Affiliated Hangzhou Hospital, Hangzhou First People's Hospital, Hangzhou, China

**Keywords:** Autophagy, p38 signaling, Hepatotoxicity, Dasatinib

## Abstract

Liver dysfunction is a common side effect associated with the treatment of dasatinib and its mechanism is poorly understood. Autophagy has been thought to be a potent survival or death factor for liver dysfunction, which may shed the light on a novel strategy for the intervention of hepatotoxicity caused by dasatinib. In this study, we show for the first time that autophagy is induced, which is consistent with the formation of liver damage. Autophagy inhibition exacerbated dasatinib-induced liver failure, suggesting that autophagy acted as a self-defense mechanism to promote survival. Oxidative stress has been shown to be an important stimulus for autophagy and hepatotoxicity. Interestingly, dasatinib increased the activity of p38, which is a critical modulator of the oxidative stress related to liver injury and autophagy. p38 silencing significantly blocked LC3-II induction and p62 reduction by dasatinib, which was accompanied by increased caspase-3 and PARP cleavage, indicating that autophagy alleviated dasatinib-induced hepatotoxicity via p38 signaling. Finally, the p38 agonist isoproterenol hydrochloride (ISO) alleviated dasatinib-induced liver failure by enhancing autophagy without affecting the anticancer activity of dasatinib. Thus, this study revealed that p38-activated autophagy promoted survival during liver injury, which may provide novel approaches for managing the clinical applications of dasatinib.

## INTRODUCTION

Although tyrosine kinase inhibitors are recognized as targeted therapies by exhibiting improved antitumor effects with lower toxicities than traditional chemotherapies, increasing clinical evidence suggests that they also have off-target effects on non-cancerous cells, which can lead to unexpected side effects.[1] As a novel, oral-administrated, and multi-targeted inhibitor of the BCR-ABL and Src family kinases, dasatinib is approved for the treatment of all chronic myelogenous leukemia (CML) and Ph-positive acute lymphocytic leukemia patients resistant or intolerant to the first-line therapy.[2] However, hepatotoxicity is a specific concern associated with dasatinib in clinic. In a phase II study conducted on 186 patients with chronic-phase CML, after 8 months of continuous dasatinib treatment, 52% and 60% of patients experienced elevated alanine aminotransferase (ALT) and aspartate aminotransferase (AST) activity, respectively.[3] A similar result is also observed in another phase II study performed on 116 patients with CML in myeloid blast crisis (MBC) or lymphoid blast crisis (LBC). After 6 months of follow-up, 8%, 4% and 8% of patients treated with dasatinib experienced grade 3 to 4 elevated ALT, AST and bilirubin activity, respectively.[4] Therefore, liver dysfunction during treatment with these medications is now receiving considerable attention, and the discovery of new defenses against dasatinib-induced liver injury may improve its clinical efficiency through ensuring the safety of long-term application of dasatinib.

Autophagy is a bulk intracellular degradation system and plays critical roles in response to stress situations.[5] In general, autophagy appears to promote survival by removing unwanted cellular substances and providing nutrients during starvation conditions. However, with increased stress, autophagy could also contribute to cell death, resulting in excessive catabolism, the misrecognition of cargo and/or commandeering of the apoptosis machinery.[6, 7] Although there is increasing evidence for the involvement of autophagy in cell survival and death during liver injury, the occurrence and role of autophagy in dasatinib-driven hepatotoxicity are unknown. Since reactive oxygen species (ROS) is one of the potential stimuli of autophagy [8], together with the fact that ROS is involved in dasatinib-induced death of rat primary hepatocytes [9], autophagy might also be activated in the liver during cancer treatment. More importantly, exploring the role and mechanism of autophagy in liver may shed light on a novel strategy for the intervention of hepatotoxicity caused by dasatinib.

p38 signaling is strongly activated by environmental and genotoxic stresses [10], and a number of documents demonstrated that p38 is a contributive factor in the development of autophagy.[11] p38-MAPK pathway has a dual role in autophagy, which depends on the type of cell and stimuli. For instance, MAPK14/p38α is reported to confer irinotecan resistance to TP53-defective cells by inducing survival autophagy.[12] Suppression of the p38-MAPK signaling pathway promotes autophagic cell death in TNFα-treated L929 cells.[13] Though the vital role of p38 in the modulation of autophagy activity has been demonstrated in regulation of various diseases such as ischemia-reperfusion [14] and myotube atrophy [15], its role in hepatotoxicity remains unclear. Also, it is unknown if the regulation of p38 activity could protect against dasatinib-induced liver damage via the modulation of autophagy.

In the present study, we observed that autophagy is induced in dasatinib-treated primary cultured hepatocytes and liver tissues. In addition, pharmacological or biological suppression of autophagy exacerbated dasatinib-induced liver injury both *in vitro* and *in vivo*, suggesting that dasatinib-induced autophagy plays a protective role against the hepatotoxic effects of this drug. Further data suggests that oxidative stress-driven MAPK signaling might contribute to dasatinib-induced autophagy. By selectively inhibiting individual branches of MAPK signaling (e.g., ERK, JNK and p38), dasatinib-induced autophagy were shown to be activated by p38. In particular, the p38 agonist isoproterenol hydrochloride (ISO) could alleviate the dasatinib-induced liver injury by enhancing autophagy without compromising the anticancer activity of dasatinib. Taken together, this study not only represents the first attempt to characterize the occurrence and role of autophagy on the dasatinib-driven hepatotoxicity both *in vitro* and *in vivo*, but also provides a new strategy for protection against dasatinib-induced hepatotoxicity through modulating the activation of p38.

## RESULTS

### Hepatotoxicity highly accompanies the anticancer activity of dasatinib

**Figure 1:**
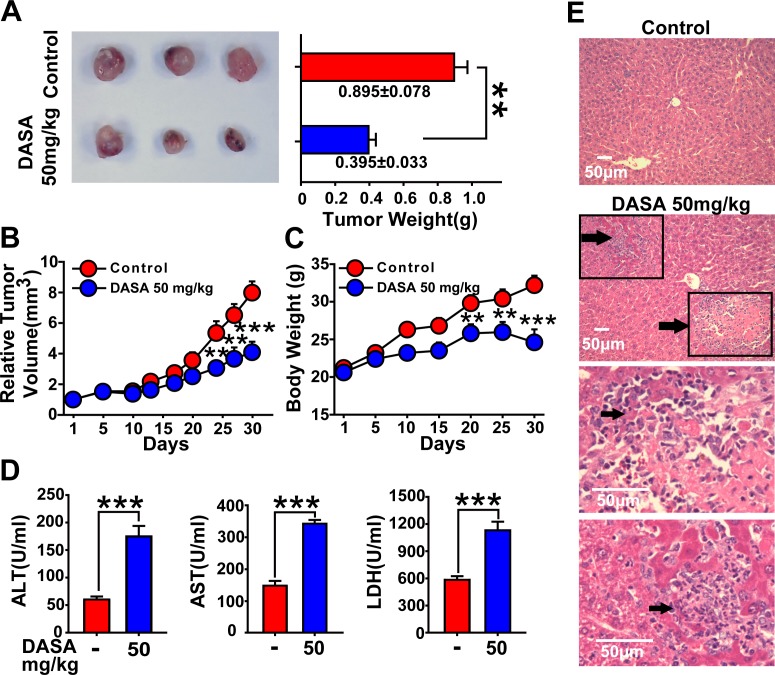
Hepatotoxicity is highly accompanied with the anticancer activity of dasatinib Nude mice transplanted with A549 human xenografts were randomly divided into 2 groups (n=3 in each group) and treated with saline or dasatinib (50 mg/kg) for 30 days. A and B. The volume and RTV of xenograft tumors. C. The body weights of each group. (D-E) Nude mice were euthanized on day 30. D. Serum was analyzed for ALT, AST and LDH levels. E. Liver sections were stained with H&E (magnification=100× or 400×, scale bar=50 μm). Black arrows denote damaged hepatocytes. Data are expressed as the mean ± SEM. Statistical analysis was performed by t-test where appropriate. **P<0.01, ***, *P*<0.001 for significant differences compared to dasatinib-treated animals. DASA=dasatinib.

Since dasatinib therapy has been examined in phase I/II studies of solid tumors (non-small cell lung cancer, prostate cancer and breast cancer) with only modest clinical activity due to its dose limitation based on liver failure (the median daily dasatinib dose was 178 mg) [16], we are encouraged to further assess the relationship between hepatotoxicity and antitumor activity in a xenograft mouse model bearing A549 lung cancer cells. Nude mice inoculated with A549 cells were divided into 2 groups, and it was found that 50 mg/kg dasatinib induced effective antitumor activity coupled with remarkable liver injury. As indicated in Fig. 1A and B, tumor growth by dasatinib treatment was significantly inhibited compared to the control group (mean tumor weight and relative tumor volume (RTV) of the dasatinib group vs. control group: *P*<0.01 and *P*<0.001). Meanwhile, the serum hepatic enzyme activities of ALT, AST and LDH were dramatically increased in dasatinib treatment group indicating elevated hepatocyte membrane permeability and cellular leakage (Fig. 1D). Furthermore, treated mice exhibited histopathological changes in the liver; in particular, 10% to 15% liver slices stained by H&E displayed necrosis (Fig. 1E). Taken together, these results clearly demonstrated that hepatotoxicity is highly accompanied with the anticancer activity of dasatinib.

### Autophagy involves in dasatinib-driven hepatotoxicity both *in vitro* and *in vivo*

**Figure 2:**
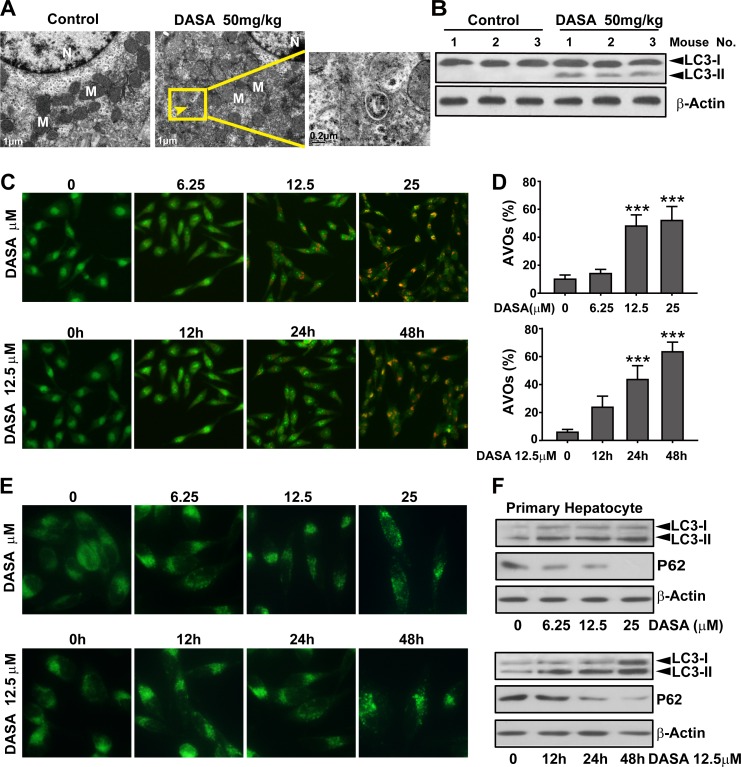
Autophagy is involved in dasatinib-driven hepatotoxicity both *in vitro* and *in vivo* A. Live tissues from the nude mice shown in Figure 1 were collected. Electron micrographs of livers from mice after dasatinib treatment (scale bars=1 μm). Autophagosomes were observed. Insets show a higher magnification view of autophagic vesicles (scale bars=0.2 μm). Yellow arrows denote autophagosomes. B. Total liver lysates were analyzed by western blot using an anti-LC3 antibody. (C-F) Hepatocytes were treated with dasatinib (12.5 μM) as indicated for 12, 24 or 48 hours or with different concentrations of dasatinib (0, 6.25, 12.5 and 25 μM) for 24 hours. C. The formation of AVOs was observed. Magnification 100×. D. The percentage of AVO development in total cells was quantified by FACS and calculated from 3 independent experiments. E. The formation of fluorescent particles were measured by MDC staining and shown as the representative results from 3 independent experiments. Magnification: 200×. F. Total cell lysates were subjected to western blot. Data are expressed as the mean ± SEM. ***, *P*<0.001 for significant differences compared to the vehicle control. DASA=dasatinib.

Autophagy serves an important role in cancer cells during dasatinib anticancer therapy and several clues of evidence suggest that it's also strongly associated with liver injury.[17] Thus, we hypothesized that autophagy might be involved in dasatinib-driven hepatotoxicity. To study whether autophagy was activated by dasatinib, liver tissues of nude mice were collected from both the vehicle control and dasatinib treatment groups. As illustrated in Fig. 2A, Transmission electron microscopic observations revealed that dasatinib treatment led to the accumulation of autophagic vacuoles in liver cells. In addition, results from western blot analysis (Fig. 2B) revealed that dasatinib increased the endogenous LC3-II levels (a widely accepted autophagy marker) in liver tissues. Collectively, these results support our hypothesis that dasatinib induces autophagy *in vivo*.

Because autophagy is a multi-phase and multi-factorial process, several approaches were applied in primary cultured rat hepatocytes to further evaluate dasatinib-induced autophagy. AO, a lysosomotropic agent, is able to stain AVOs and offers a rapid and quantitative method to measure the induction of autophagy.[17] We evaluated AVOs in dasatinib-treated primary hepatocyte cells by AO staining followed by FACS analysis. As shown in Fig. 2C and D, a time- and concentration-dependent increase in AVOs were observed in dasatinib-treated primary hepatocyte cells. MDC is an autofluorescent agent that is specifically concentrated in autophagolysosomes.[17] As shown in Fig. 2E, treatment with dasatinib induced the accumulation of MDC in cytoplasmic vacuoles in a time- and concentration-dependent manner. To confirm that the observed vesicles were indeed related to autophagy, we examined LC3 modification and p62 expression by western blot analysis. And the results confirmed the conversion of LC3 (LC3-I to LC3-II) and dramatic degradation of p62 after dasatinib treatment in primary cultured rat hepatocytes (Fig. 2F), indicating that dasatinib induced autophagic flux. Once again, these results demonstrate that autophagy is activated in dasatinib-induced liver injury.

**Figure 3:**
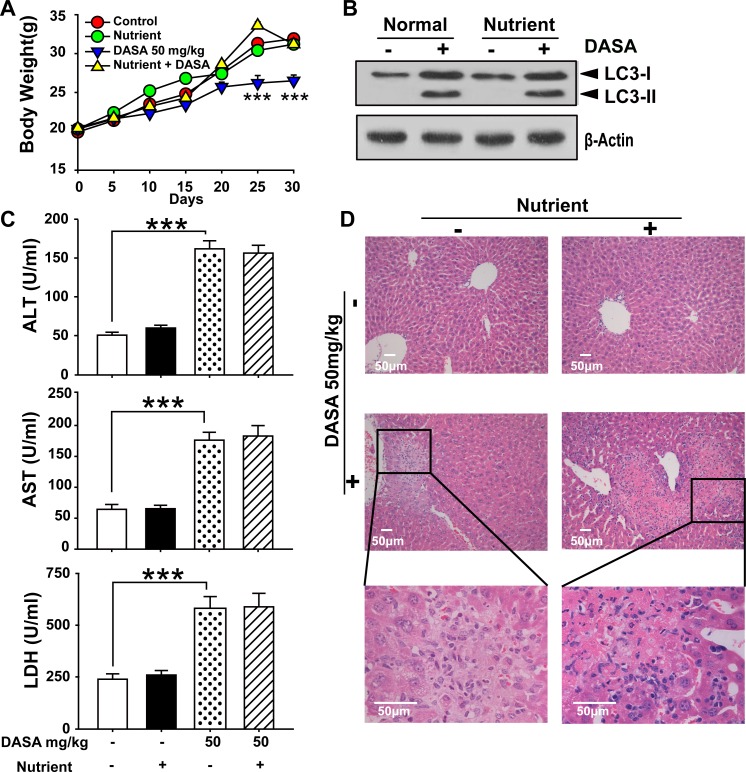
Supplemental nutrients had no effect on the autophagy and liver injury of dasatinib ICR mice were randomly divided into 4 groups (n=6 mice) and treated with supplemental nutrients (10 ml/kg), dasatinib (50 mg/kg) or both for 30 days. A. Average body weight. B. Serum was analyzed for ALT, AST and LDH levels. C. Total liver lysates were analyzed by western blot using antibodies against cleaved caspase-3, cleaved PARP, LC3, p-p38 and β-actin. D. Liver sections were stained with H&E for histopathological analysis (magnification=100× or 400×, scale bar=50 μm). Black arrows denote necrotic hepatocytes. ***, *P*<0.001 for significant differences compared to the vehicle control. DASA=dasatinib.

**Figure 4:**
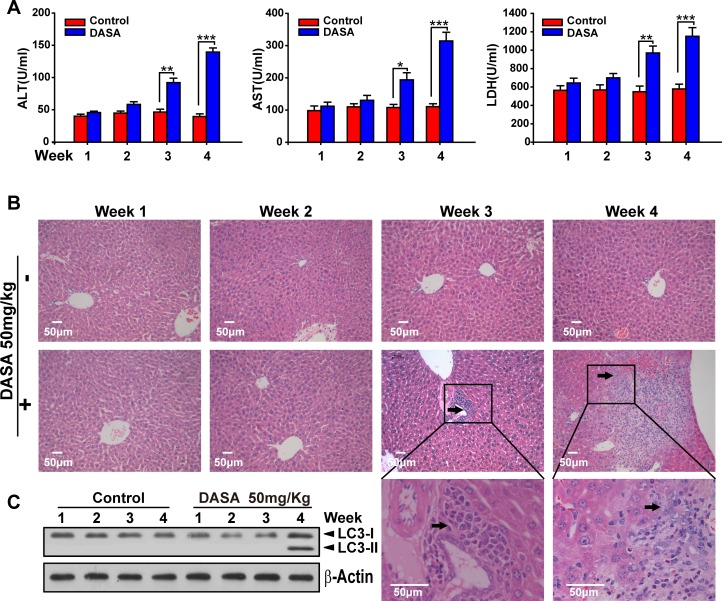
Dasatinib induced the liver injury and autophagy sequence ICR mice were randomly divided into two groups (n=16) and were treated either with saline or dasatinib (50 mg/kg) for 4 weeks, and sacrificed 4 of 16 each group every week. A. Serum was analyzed for ALT, AST and LDH level. B. Liver sections were stained with hematoxylin and eosin for histopathological analysis (magnification=×100 or 400, scale bar=50 μm). Black arrows denote necrotic hepatocytes. C. Protein extracts of liver tissues were immunoblotted with the specified antibodies for LC3 and β-actin. Data are expressed as the mean ±SEM. Statistical analysis was performed by t-test where appropriate. DASA = dasatinib.

Since autophagy serves as an alternative energy source for sustaining cellular function during starvation and the weights of dasatinib-treated mice were also decreased [18], it is possible that the autophagy observed in our study was the result of reduced calorie intake. To detect whether autophagy was directly activated by dasatinib, we compared the food intake between the treated and non-treated groups and found no significant difference (Supporting Fig.1). Furthermore, we added supplemental nutrients to the dasatinib group of which the body weights were approximately the same as the control group. It was shown that the dasatinib-induced hepatotoxicity and autophagy were unchanged (Fig.3). In addition, to clarify the hepatotoxicity and autophagy sequence, daily treatments of dasatinib were administered for 1 to 4 weeks in healthy mice. We found that dasatinib-induced liver dysfunction began at 3 weeks, according to H&E staining of liver sections and serum ALT, AST and LDH activity measurements, while autophagy was only activated after 4 weeks of treatment (Fig. 4). These results indicated that autophagy was induced after dasatinib treatment.

### Dasatinib-induced autophagy protects against its hepatotoxicity both *in vitro* and *in vivo*

**Figure 5:**
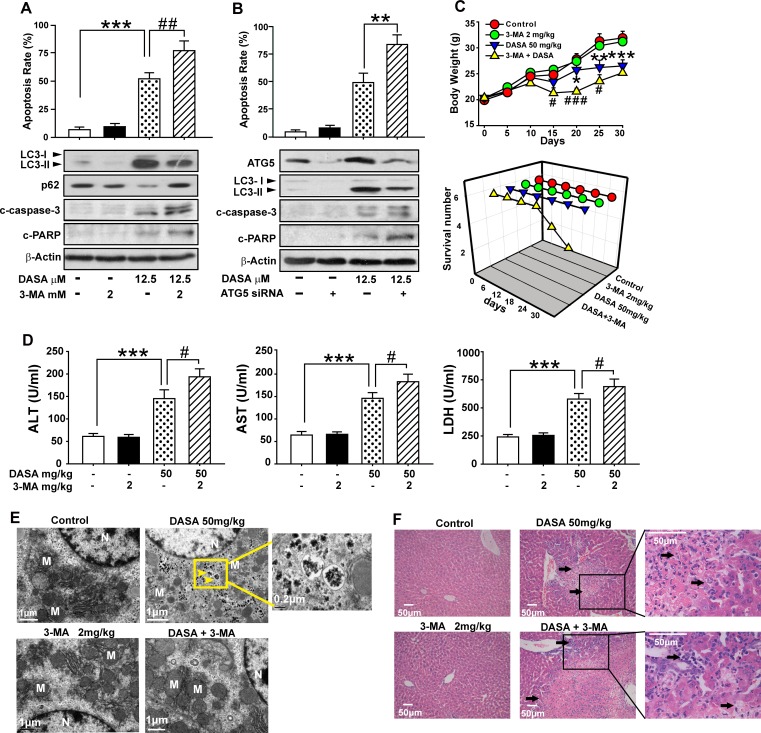
Dasatinib-induced autophagy protects against its hepatotoxicity both *in vitro* and *in vivo* A. Hepatocytes were treated with dasatinib (12.5 μM), 3-MA (2 mM) or both for 24 hours. B. Hepatocytes were transfected with 100 nM nontargeting siRNA (control) or siRNA targeting Atg5 (siRNA), treated with dasatinib (12.5 μM) for another 24 hours. Annexin V and PI-staining cells were quantified by FACS. Total cell lysates were subjected to western blot. (C-F) ICR mice were randomly divided into 4 groups (n=6 per group) and treated with 3-MA (2 mg/kg), dasatinib (50 mg/kg) or both for 30 days, and were euthanized on day 30. C. The body weight and survival rate of mice in each group was collected. D. Serum was analyzed for ALT, AST and LDH levels.E. Electron micrographs of livers from mice after treatment (scale bars=1 μm). Autophagosomes were observed. Insets show a higher magnification view of autophagic vesicles (scale bars=1 μm). Yellow arrows denote autophagosomes. F. Liver sections were stained with H&E (magnification=100× or 400×, scale bar=50 μm). Black arrows denote damaged hepatocytes. Data are expressed as the mean ± SEM. *, *P*<0.05, **, *P*<0.01, ***, *P*<0.001 for significant differences compared to the vehicle control. ^#^, *P*<0.05, ^##^, *P*<0.01 for significant differences compared to the dasatinib group. DASA=dasatinib.

To address the role of autophagy in dasatinib-induced hepatotoxicity, the hepatocytes were pretreated for 24 hours with 3-MA, the specific inhibitor of autophagosome formation, followed by dasatinb exposure. We found that apoptosis induction by dasatinib was markedly enhanced in the presence of 3-MA, as determined by Annexin V and PI staining. In addition, consistent with the data from FACS, pretreatment with 3-MA also increased the dasatinib-induced cleavage of PARP and caspase-3, which serve as markers for apoptosis, suggesting that dasatinib-induced hepatocyte apoptosis was augmented by the inhibition autophagy (Fig. 5A).

Given that Atg5, a key component of the Atg12-Atg5 conjugation complex, is required for the formation of autophagosome [19], a small interfering RNA (siRNA) specifically targeting ATG5 was employed to confirm the effects of autophagy inhibition on dasatinib-induced apoptosis. As shown in Fig. 5B, Atg5 expression was knocked down by RNA interference in primary cultured rat hepatocytes. As expected, cells transfected with Atg5 siRNA showed reduced levels of LC3-II accumulation after dasatinib treatment when compared to a random control. Of note, the apoptosis rates of hepatocyte cells exposed to dasatinib significantly increased in the Atg5 knockdown group compared to the negative control group. These results demonstrated that the blockade of autophagy subsequently enhanced the apoptosis associated with dasatinib treatment.

To test whether this protection effect could be reproduced *in vivo*, ICR mice were treated with dasatinib in the presence or absence of 3-MA. TEM observations confirmed that treatment with 3-MA eliminated dasatinib-induced autophagy and resulted in characteristic cytoplasm vacuolation (Fig. 5E). More importantly, 3-MA treatment aggravated the serum levels of ALT, AST and LDH induced by dasatinib (Fig. 5D). In particular, 2 out of 6 mice died after treated with 3-MA and dasatinib (Fig. 5C). By H&E staining, centrilobular necrosis was evident in mouse livers after dasatinib treatment, which was further exacerbated by 3-MA treatment (Fig. 5F). Therefore, these *in vivo* data further confirmed that dasatinb-induced autophagy protected against its hepatotoxicity.

### The activation of p38 is required for dasatinib-induced autophagy in hepatocytes

**Figure 6:**
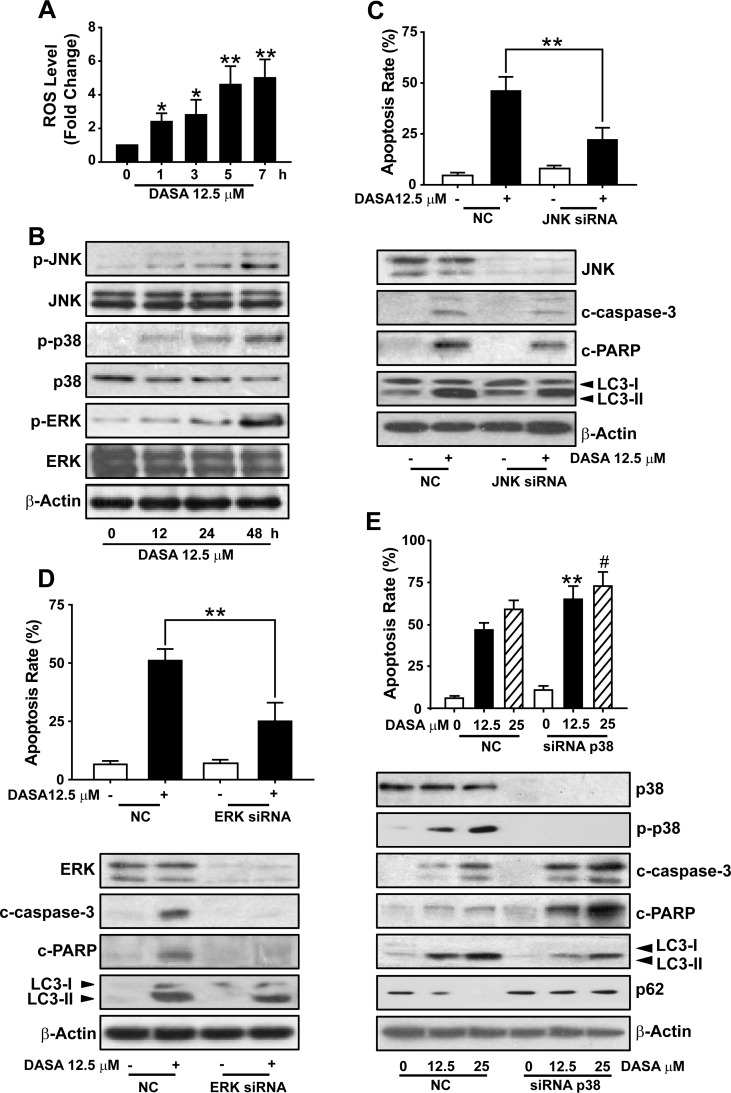
The activation of p38 is required for dasatinib-induced autophagy in hepatocytes A. ROS level was detected using H2DCFDA and FACS following dasatinib treatment for various times (0-7 h). B. Protein expression levels were detected by western blot. C-E. Hepatocytes were transfected with 100 nM of non-targeting siRNA (control) or a siRNA targeting JNK (C), ERK (D) or p38 (E) and treated with dasatinib for an additional 24 hours. Apoptosis rate was determined by FACS after Annexin V and PI staining. The protein extracts were subjected to western blot. *, *P*<0.05, **, *P*<0.01 for significant differences compared to the vehicle control.^#^, *P*<0.05 for significant differences compared to the dasatinib group. DASA=dasatinib.

Considering the protective role of autophagy in dasatinib-driven hepatotoxicity, we were next encouraged to address the mechanism of dasatinib-induced autophagy in the liver. Given that (i) the accumulation of ROS has been proposed to induce autophagy [18], (ii) the ROS level in dasatinib-treated hepatocytes was increased significantly compared to untreated cells after treatment with 12.5 μM dasatinib for various time periods (0-7 hours) (Fig. 6A), (iii) MAPKs are critical regulatory proteins of the hepatic response to oxidative stress that regulate cell death, injury, proliferation and differentiation [20], we investigated the activation of three subfamilies of MAPKs (ERK, JNKs and p38) by dasatinib in primary cultured rat hepatocytes. As illustrated in Fig. 6B, despite the unchanged total protein expression level of p38, JNK and ERK during dasatinib treatment, dasatinib induced a sharp increase in the phosphorylation of p38, JNK and ERK, indicating that dasatinib might induce autophagy through the activation of the MAPKs pathway.

To better illustrate the specific roles of the three individual branches of MAPKs in dasatinib-induced autophagy, siRNA against JNK, ERK and p38 were used. As shown in Fig. 6D, JNK siRNA transfection in these cells led to a marked decrease in JNK expression, and the dasatinib-induced cleavage of apoptotic proteins such as caspase-3 and, whereas failed in affecting the conversion of LC3. Meanwhile, the expression levels of apoptotic proteins were also significantly decreased in the ERK knockdown group compared to the negative control group, without altering the degree of autophagy caused by dasatinib. These results suggested that dasatinib might induce autophagy through a JNK- or ERK-independent pathway.

Next, we evaluated the role of p38 in dasatinib-induced autophagy. As shown in Fig. 6F, the siRNA-mediated loss of p38 expression was verified by western blot. Interestingly, the inhibition of p38 expression by siRNA stimulated dasatinib-induced hepatocyte cell death. Furthermore, silencing of p38 in primary cultured rat hepatocytes dramatically blocked the LC3-II induction and p62 reduction by dasatinib, which was accompanied with the increase of caspase-3 and PARP cleavage. Taken together, these results not only suggest that the activation of p38 is required for dasatinib-induced autophagy in hepatocytes, but also proposed that p38 could be considered as a potential target to ameliorate dasatinib-induced hepatotoxicity.

### Induction of p38-regulated autophagy by isoproterenol hydrochloride attenuates dasatinib-induced hepatoxicity

**Figure 7:**
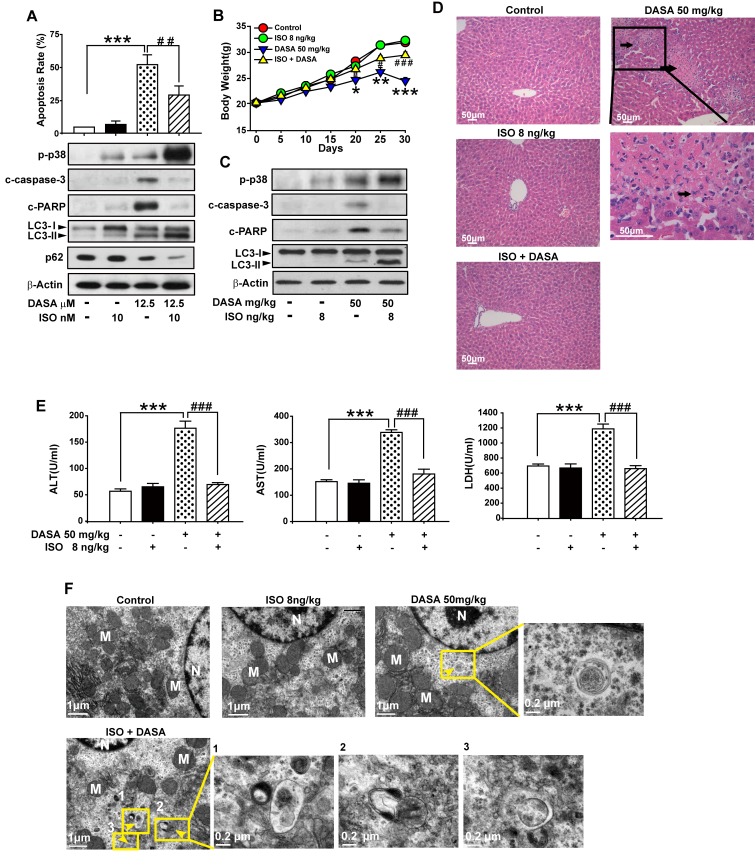
Induction of p38-regulated autophagy by isoproterenol hydrochloride attenuated dasatinib-induced hepatoxicity *In vitro* study, hepatocytes were treated with dasatinib (12.5 μM), ISO (10 nM), or both for 24 hours. A. Apoptosis rate was determined by FACS. Protein lysates of cells were subjected to western blot. (B-F) *In vivo* study, ICR mice were randomly divided into 4 groups (n=6 per group) and treated with ISO (8 ng/kg), dasatinib (50 mg/kg) or both for 30 days. All the mice were euthanized on day 30. B. The body weight of every groups. C. Total liver lysates were analyzed by western blot. D. Liver sections were stained with H&E (magnification=100× or 400×, scale bar=50 μm). Black arrows denote damaged hepatocytes. E. Serum was analyzed for ALT, AST and LDH levels. F. Electron micrographs of livers from mice after treatment (scale bars=1 μm). Autophagosomes were observed. Insets show a higher magnification view of autophagic vesicles (scale bars=0.2 μm). Yellow arrows denote autophagosomes. Data are expressed as the mean ± SEM. *, *P*<0.05, **, *P*<0.01, ***, *P*<0.001 for significant differences compared to the vehicle control. ^##^, *P*<0.01, ^###^*P*<0.001 for significant differences compared to the dasatinib group. DASA=dasatinib. ISO=isoproterenol hydrochloride.

Recently, many studies have established that the concentration of isoproterenol hydrochloride (ISO) used for the activation of p38 is far below the one for cardiovascular disease.[21] Here, we administrated 10 nM ISO (only sufficient for the activation p38 protein) together with dasatinib in hepatocyte cells and found that ISO down-regulated dasatinib-induced primary cultured rat hepatocyte death by enhancing autophagy (Fig. 7A). Western blot analysis revealed a significant increase in the amount of LC3-II, as well as a reduction of p62 after dasatinib combination with ISO compared to single treatment of dasatinib. As expected, the increase of autophagy induction was also accompanied with the decrease of caspase-3 and PARP cleavage. Collectively, these results suggest that the enhancement of p38 activity by ISO may protect against dasatinib-induced liver damage via the induction of autophagy.

To determine whether induction of p38-regulated autophagy by ISO could attenuate dasatinib-induced hepatoxicity *in vivo*, ICR mice were treated with dasatinib in the absence or presence of ISO. Compared to the control and ISO groups, ISO (8 ng/kg) in combination with dasatinib almost totally reversed the dasatinib-mediated increase of ALT, AST and LDH (Fig. 7E). In addition, centrilobular necrosis were evident in the livers of mice treated with dasatinib by H&E staining, whereas this type of liver damage was virtually absent after ISO treatment (Fig. 7D). Furthermore, ISO combination decreased the level of activated caspase-3 compared to dasatinib treatment alone, whereas p-p38 was increased as expected (Fig. 7C). These results indicated that ISO could protect against dasatinib-induced liver damage *in vivo*. To confirm that ISO attenuated dasatinib-induced liver failure by activating p38-mediated autophagy, TEM and western blotting were performed. Treatment with ISO enhanced dasatinib-induced autophagy by leading to the accumulation of autophagolysosomes and LC3-II (Fig. 7C and F). These results demonstrated that ISO attenuated dasatinib-induced liver injury by stimulating p38-dependent autophagy *in vivo*. More importantly, the dose of ISO (8 ng/kg/day) used to reduce dasatinib-induced hepatotoxicity in ICR mice was approximately 1/(2×10^8^) of its LD50 [22], indicating that ISO has potential clinical application for treating dasatinib-induced liver failure.

### Isoproterenol hydrochloride does not affect the anticancer activity of dasatinib

**Figure 8:**
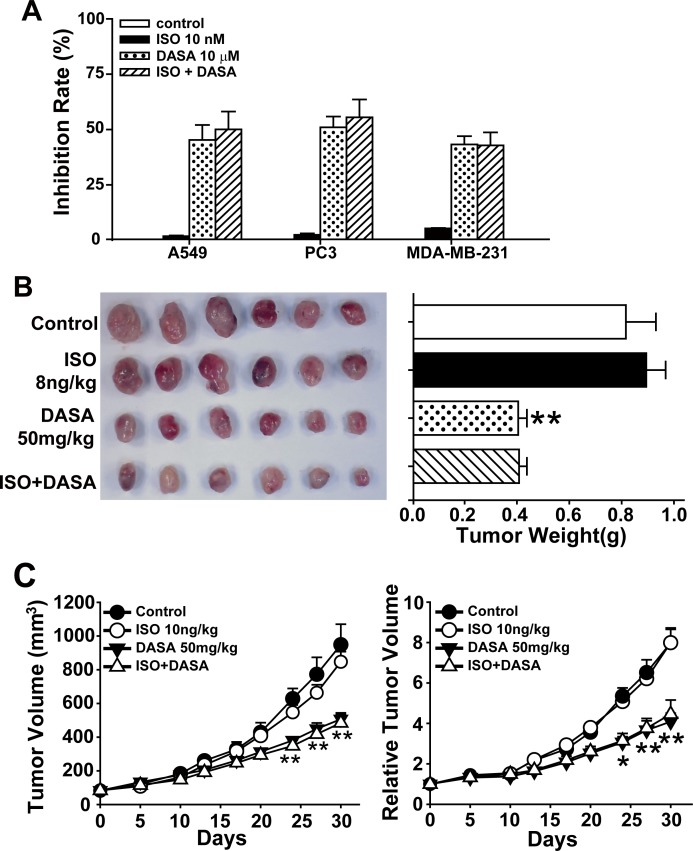
Isoproterenol hydrochloride does not affect the anticancer activity of dasatinib A. A549, PC3 and MDA-MB-231 cells were treated with ISO (10 nM), dasatinib (12.5 μM) or both for 24 hours. Apoptosis rate was determined by FACS following Annexin V and PI staining. (B-C) Nude mice transplanted with A549 human xenografts were randomly divided into 4 groups (n=6 per group) and treated with ISO (8 ng/kg), dasatinib (50 mg/kg) or both for 30 days. All the mice were euthanized on day 30. B. Volume of xenograft tumors. C. RTV. *, *P*<0.05, **, *P*<0.01 for significant differences compared to the vehicle control. DASA=dasatinib. ISO=isoproterenol hydrochloride.

Protective factors against hepatotoxicity, including vitamin C, glutathione, tiopronin and N-acetylcysteine (NAC) [23-25], have been shown to reduce the anticancer activity of anticancer drugs. Therefore, as we hope to promote the clinical use of dasatinib in solid tumors, we next examined whether ISO could influence the antitumor properties of dasatinib. We first detected apoptosis by Annexin V and PI staining in the non-small cell lung cancer cell line A549, the prostate cancer cell line PC3 and the breast cancer cell line MDA-MB-231. All three cell lines were treated with 10 nM ISO, 10 μM dasatinib or their combination for 24 hours. As shown in Fig. 8A, the proportion of apoptotic cells after dasatinib treatment did not decrease when the cells were co-treated with ISO. To further characterize the anticancer efficacy of ISO and dasatinib combination treatment, the *in vivo* activity of ISO and dasatinib was tested in the A549 xenograft nude mouse model. As shown in Fig. 8B and C, the intraperitoneal administration of ISO at a dose of 8 ng/kg each day for 30 days showed no significant difference in the mean tumor weight or RTV compared to the dasatinib-alone group. Thus, ISO has potential clinical application for mitigating the liver injury resulting from dasatinib treatment without influencing its anticancer activity.

## DISCUSSION

Autophagy is important for many physiological and pathological processes. Much of the pioneering work in the autophagy field has been conducted on the liver or isolated hepatocytes, including determination of the morphology and membrane composition of autophagosomes, characterization of the kinetics of protein degradation and regulation of autophagy by hormones and amino acids.[26, 27] However, it remains unclear whether autophagy fundamentally acts as a cell survival or cell death pathway during liver injury.[18] In hepatic cells, the formation of autophagosomes and autophagolysosomes for degrading cellular components for energy production in response to nutrient deprivation is an underlying protective response against ischemia-reperfusion injury.[28] In contrast, concanavalin A, a lectin with mannose specificity that can induce acute hepatic inflammation, triggers autophagy-related necrotic hepatocyte death through IRGM1-mediated lysosomal membrane disruption.[29] In this study, we first determined that autophagy was activated by dasatinib in liver, and dasatinib-induced hepatoxicity is the reason of autophagy, thus indicating that autophagy is likely an important mechanism for dasatinib-induced liver injury. Of note, we also presented evidence that loss of autophagy enhanced liver injury after dasatinib treatment, suggesting that the activation of autophagy functions to protect hepatocytes in a similar manner as in the ischemia-reperfusion model.

Autophagy is tightly regulated by a number of pathways, of which the most extensively studied involves the protein kinase mTOR (mammalian target of rapamycin), which negatively regulates autophagy. Inhibition of this pathway with the drug rapamycin enhances autophagy.[30, 31] However, in our study, we found the expression of phospho-mTOR (ser2448) as well as the levels of phosphorylated p70S6 kinase and 4E-BP1 was almost same in primary hepamyocytes after exposure to dasatinib(Supporting Fig.2). In addition, rapamycin did not reduce the hepatotoxicity of dasatinib. These results suggested dasatinib-induced autophagy via an mTOR-independent pathway.

Recently, the themes of oxidative stress and autophagy have been brought together. A diverse range of stimuli that induce both ROS and autophagy have been described and autophagy induction by these agents is antagonized by antioxidants.[32, 33] Meanwhile, antioxidants such as NAC inhibited mTOR signaling and diminished oxidative stress mediated damage in several diseases by activating autophagy.[34, 35] For this reason, we tested whether dasatinib also increase oxidative stress, and found ROS was up-regulated. However, ROS pan scavenger NAC blocked both autophagy and hepatotoxicity induced by dasatinib(Supporting Fig.3). In addition, NAC even reduced the anticancer activity of dasatinib(Supporting Fig.4), suggesting that these pan oxidative stress inhibitors may be limited in the context of liver injury caused by dasatinib. It is important to discover the mechanism that autophagy protect against dasatinib-induced hepatotoxicity.

p38 is a critical factor in regulating cell death and autophagy, and increasing clinical reports have shown that activation of the p38-MAPK pathway serves as an important cellular protective mechanism in patients with acute and chronic liver failure.[36] Furthermore, there are abundant evidence demonstrating the role of p38 in damage following ischemia-reperfusion. For example, p38 is phosphorylated and activated several minutes after reperfusion, and p38 activation is associated with the induction of apoptosis and necrosis. In addition, the role of p38 in the modulation of autophagy activity has been demonstrated in recent studies. For example, the inhibition of p38 activity was shown to attenuate myotube atrophy by inhibiting the expression of autophagy-related genes *in vitro*. Models of dasatinib-induced hepatotoxicity have further shown that p38 activation is typically associated with autophagy induction. Of note, dasatinib-induced autophagy can be suppressed by p38 inhibition, suggesting that p38 activation is an essential step for the stimulation of dasatinib-mediated autophagy. Furthermore, inhibition of p38 activity also enhanced dasatinib-induced liver injury, which demonstrated that p38 might act as a guardian against dasatinib-induced hepatotoxicity. Therefore, targeting p38 could provide a new strategy for protection against dasatinib-induced hepatotoxicity.

ISO was recently reported to act as a specific p38 agonist at low doses in the repair of tissue injuries.[21] Here, we found that stimulating p38 activity by ISO at a low concentration could enhance dasatinib-induced autophagy, which mitigated liver dysfunction both *in vitro* and *in vivo*. Compared to other protective agents against liver dysfunction, ISO did not reduce the anticancer activity of dasatinib in solid tumor. It should be particularly noted that the dose of ISO used to protect against dasatinib-induced liver injury was 1.25×10^−7^ of the dose used to treat bradycardia in a mouse model, implying that there might be no obvious toxicity. Importantly, ISO has a long history of clinical application, indicating that it may be a potential therapeutic drug for treating dasatinib-induced liver injury.

In conclusion, we report a novel protective role for autophagy against dasatinib-induced hepatotoxicity in both liver tissues and primary cultured rat hepatocytes. Furthermore, we demonstrated that autophagy regulates liver failure via a mechanism that involves the activation of p38 signaling. Most importantly, the p38 agonist ISO alleviated dasatinib-induced liver failure at a low dose by enhancing autophagy without affecting the anticancer activity of dasatinib both *in vitro and in vivo*. Thus, this study revealed for the first time that p38 modulate autophagy during dasatinib-stimulated hepatotoxicity. Our findings bolster the view that p38-activated autophagy promotes survival during liver disease, which may provide novel approaches for managing the clinical applications of dasatinib. These results may also have implications for treatment with other anticancer drugs.

## MATERIALS AND METHODS

### Reagents

Dasatinib (purity: 99.1%) was a generous gift from Shanghai Institute of Materia Medica, Chinese Academy of Sciences (Shanghai, China), and was dissolved in dimethyl sulfoxide (DMSO) (50.0 mM stock solution) and stored at −20°C. The stock solution was freshly diluted to the specific concentration with growth medium before use. Isoprenaline Hydrochloride (ISO) was purchased from Sigma Chemical Co. (St. Louis, MO), and was stored at 4°C, The stock solution was freshly diluted to the specific concentration with growth medium before use. DMSO, propidium iodide (PI), acridine orange (AO), 3-methyl adenine (3-MA) and monodansylcadaverine (MDC) were purchased from Sigma Chemical Co. (St. Louis, MO). Supplemental nutrition was purchased from Fresenius Kabi Deutschland GmbH. MTT was dissolved in Dulbecco's Modified Eagle's Medium (DMEM) to make a 5 mg/ml solution. The mitochondrial fluorescent probe 2′,7′-dichlorodihydrofluorescein diacetate (H2DCFDA) were purchased from Invitrogen (CA, USA). Stock solutions of H2DCFDA (10.0 mM) were dissolved in DMSO and then stored at −20 °C. The primary antibodies against β-actin, ERK, p38, JNK, and HRP-labeled secondary anti-goat, anti-mouse and anti-rabbit antibodies from Santa Cruz Biotech (Santa Cruz, CA); LC3, cleaved caspase-3, cleaved PARP, p-JNK (Thr183/Tyr185), p-ERK(Ser70) and p-p38(Thr180/Tyr182) from Cell Signaling Technology (Beverly, MA).

### Cell Lines and Hepatocyte Preparation

All of the cell lines were purchased from Shanghai Institute of Biochemistry and Cell Biology (Shanghai, China) and the culture media were described previously.[37]

Hepatocytes were isolated from Sprague-Dawley male rats, for details please refer to supplementary data.

### Cytotoxicity Assay

Hepatocytes were seeded in 96-well plates (3 × 10^4^/well). After treatment with varying concentrations of dasatinib for 48 h, viable cells were determined using MTT assay. MTT was added (30.0 μl/well), and plates were incubated for a further of 4h at 37°C. The purple formazan crystals were dissolved in 100 μl DMSO. After the crystal dissolved, the plates were read on an automated microplate spectrophotometer (Thermo Multiskan Spectrum, Thermo Electron Corporation, USA) at 570 nm.

### Animal Treatment and Drug Administration

ICR mice were supplied by the Shanghai Laboratory Animal Center, Chinese Academy of Sciences and female BALB/c nude mice were purchased from National Rodent Laboratory Animal Resource (Shanghai, China). The animal treatment and drug administration were performed as described in supplementary data.

### Measurement of ALT, AST and LDH Leakage

Serum hepatic enzyme activity of ALT, AST and LDH was determined by full-automatic biochemical detect machine (Cobas c 311, Roche Diagnostics GmbH, Germany) using specific detective kits in the last day after last treatment.

### Analysis of Apoptosis by PI Staining

The sub-G1 analysis after Propidium iodide (PI) staining was employed to assess the apoptosis. Cells (3 × 10^5^/well) were seeded into 6-well plates and exposed to the compounds for the indicated time, and then harvested and washed with PBS, fixed with pre-cooled 70% ethanol at 4 °C. Staining went along in PBS containing 40 μg/ml RNaseA at 37 °C and 10 μg/ml PI in dark at room temperature for 30 min. For each sample 2 × 10^4^ cells were collected and analyzed using an FACS-Calibur cytometer (Becton Dickinson, USA).

### Western Blot Analysis

Western blot was performed as described before.[37]

### Acridine Orange and Monodansylcadaverine Staining

Acridine orange (AO) flow cytometry and Monodansylcadaverine (MDC) staining were used to detect the development of acidic vesicular organelles as described before [37].

### Transmission Electron Microscopic Analysis

Livers were fixed with 3% glutaraldehyde in 0.05 M sodium cacodylate buffer at pH 7.2, post-fixed with 2% osmic acid, epon-embedded, sectioned at a thickness of 1 lm, and stained with toluidine blue. Ultrathin sections were observed using a PHILIPS TECNA110 electron microscope.

### Histopathological Analysis

Liver samples were fixed in 10% phosphate-buffered formalin and embedded in paraffin. Sections were stained with hematoxylin and eosin for histopathological analysis. The sections of caudate lobe (CL), right liver lobe (RLL), median lobe (ML) and the left lateral lobe (LLL) were examined under light microscopy.

### Transfection and RNAi

The siRNA for p38, ERK and JNK were purchased from Santa Cruz Biotechnology (Santa Cruz, USA). The transfection was performed using Opti-MEM, siRNA and oligofectamine (Invitrogen, USA) according to the manufacturer's recommendations, 24 hours prior to the drug treatment. After 24 hours of exposure to dasatinib for indicated times, treated or untreated cells were collected for further analysis.

### Statistical Analysis

The data were expressed as means ± SEM. Two tailed student's t-tests were used to determine the significance of differences between the experiment conditions. *Indicates the values are significantly different than the control (*P<0.05, **P<0.01, ***P<0.001). #Indicates the values are significantly different than the dasatinib (#P< 0.05, ## P<0.01, ###P<0.001). DASA = dasatinib.

## SUPPLEMENTARY MATERIAL FIGURES



## Abbreviations

3-MA: 3-methyladenine
ALT: alanine aminotransferase
AST: aspartate aminotransferase
LDH: lactate dehydrogenase
DASA: dasatinib
TEM: Transmission electron microscopy
H&E: hematoxylin and eosin
i.g: intragastric
i.p: intraperitoneal;
LC3: light chain 3
AO: acridine orange
MDC: monodansylcadaverine
PI: propidium iodide
ISO: isoproterenol hydrochloride
ROS: reactive oxygen species
SEM: standard error of the mean
RTV: relative tumor volume
TUNEL: terminal dUTP nick-end labeling

## References

[1] Hartmann JT, Haap M, Kopp HG, Lipp HP (2009). Tyrosine kinase inhibitors - a review on pharmacology, metabolism and side effects. Current drug metabolism.

[2] Bonvin A, Mesnil A, Nicolini FE, Cotte L, Michallet M, Descotes J, Vial T (2008). Dasatinib-induced acute hepatitis. Leukemia & lymphoma.

[3] Jabbour E, Deininger M, Hochhaus A (2011). Management of adverse events associated with tyrosine kinase inhibitors in the treatment of chronic myeloid leukemia. Leukemia.

[4] Rea D, Bergeron A, Fieschi C, Bengoufa D, Oksenhendler E, Dombret H (2008). Dasatinib-induced lupus. Lancet.

[5] Yu L, McPhee CK, Zheng L, Mardones GA, Rong Y, Peng J, Mi N, Zhao Y, Liu Z, Wan F, Hailey DW, Oorschot V, Klumperman J, Baehrecke EH, Lenardo MJ (2010). Termination of autophagy and reformation of lysosomes regulated by mTOR. Nature.

[6] Komatsu M, Kurokawa H, Waguri S, Taguchi K, Kobayashi A, Ichimura Y, Sou YS, Ueno I, Sakamoto A, Tong KI, Kim M, Nishito Y, Iemura S, Natsume T, Ueno T, Kominami E (2010). The selective autophagy substrate p62 activates the stress responsive transcription factor Nrf2 through inactivation of Keap1. Nature cell biology.

[7] Ni HM, Bockus A, Boggess N, Jaeschke H, Ding WX (2012). Activation of autophagy protects against acetaminophen-induced hepatotoxicity. Hepatology.

[8] Chen Y, Azad MB, Gibson SB (2009). Superoxide is the major reactive oxygen species regulating autophagy. Cell death and differentiation.

[9] Xue T, Luo P, Zhu H, Zhao Y, Wu H, Gai R, Wu Y, Yang B, Yang X, He Q (2012). Oxidative stress is involved in Dasatinib-induced apoptosis in rat primary hepatocytes. Toxicology and applied pharmacology.

[10] Loesch M, Chen G (2008). The p38 MAPK stress pathway as a tumor suppressor or more?. Frontiers in bioscience : a journal and virtual library.

[11] Webber JL (2010). Regulation of autophagy by p38alpha MAPK. Autophagy.

[12] Paillas S, Causse A, Marzi L, De Medina P, Poirot M, Denis V, Vezzio-Vie N, Espert L, Arzouk H, Coquelle A (2012). MAPK14/p38a confers Irinotecan resistance to p53-defective cells by inducing survival autophagy. Autophagy.

[13] Ye Y-C, Yu L, Wang H-J, Tashiro S-i, Onodera S, Ikejima T (2010). TNFα-induced necroptosis and autophagy via supression of the p38-NF-κB survival pathway in L929 cells. Journal of pharmacological sciences.

[14] Kobayashi M, Takeyoshi I, Yoshinari D, Matsumoto K, Morishita Y (2002). P38 mitogen-activated protein kinase inhibition attenuates ischemia-reperfusion injury of the rat liver. Surgery.

[15] McClung JM, Judge AR, Powers SK, Yan Z (2010). p38 MAPK links oxidative stress to autophagy-related gene expression in cachectic muscle wasting. American journal of physiology Cell physiology.

[16] Johnson FM, Bekele BN, Feng L, Wistuba I, Tang XM, Tran HT, Erasmus JJ, Hwang LL, Takebe N, Blumenschein GR, Lippman SM, Stewart DJ (2010). Phase II study of dasatinib in patients with advanced non-small-cell lung cancer. Journal of clinical oncology : official journal of the American Society of Clinical Oncology.

[17] Hu YL, Jahangiri A, Delay M, Aghi MK (2012). Tumor cell autophagy as an adaptive response mediating resistance to treatments such as antiangiogenic therapy. Cancer research.

[18] Scott RC, Schuldiner O, Neufeld TP (2004). Role and regulation of starvation-induced autophagy in the Drosophila fat body. Developmental cell.

[19] Williams RA, Smith TK, Cull B, Mottram JC, Coombs GH (2012). ATG5 is essential for ATG8-dependent autophagy and mitochondrial homeostasis in Leishmania major. PLoS pathogens.

[20] Johnson GL, Lapadat R (2002). Mitogen-activated protein kinase pathways mediated by ERK, JNK, and p38 protein kinases. Science.

[21] Moule SK, Denton RM (1998). The activation of p38 MAPK by the beta-adrenergic agonist isoproterenol in rat epididymal fat cells. FEBS letters.

[22] Ha YM, Ham SA, Kim YM, Lee YS, Kim HJ, Seo HG, Lee JH, Park MK, Chang KC (2011). β< sub> 1</sub>-Adrenergic receptor-mediated HO-1 induction, via PI3K and p38 MAPK, by isoproterenol in RAW 264.7 cells leads to inhibition of HMGB1 release in LPS-activated RAW 264.7 cells and increases in survival rate of CLP-induced septic mice. Biochemical pharmacology.

[23] Kalender S, Uzun FG, Durak D, Demir F, Kalender Y (2010). Malathion-induced hepatotoxicity in rats: the effects of vitamins C and E. Food and chemical toxicology : an international journal published for the British Industrial Biological Research Association.

[24] Saito C, Zwingmann C, Jaeschke H (2010). Novel mechanisms of protection against acetaminophen hepatotoxicity in mice by glutathione and N-acetylcysteine. Hepatology.

[25] Weng CJ, Chen MJ, Yeh CT, Yen GC (2011). Hepatoprotection of quercetin against oxidative stress by induction of metallothionein expression through activating MAPK and PI3K pathways and enhancing Nrf2 DNA-binding activity. New biotechnology.

[26] Rautou PE, Mansouri A, Lebrec D, Durand F, Valla D, Moreau R (2010). Autophagy in liver diseases. Journal of hepatology.

[27] Saberi B, Ybanez MD, Johnson HS, Gaarde WA, Han D, Kaplowitz N (2014). Protein kinase C (PKC) participates in acetaminophen hepatotoxicity through c-jun-N-terminal kinase (JNK)-dependent and -independent signaling pathways. Hepatology.

[28] Wang JH, Ahn IS, Fischer TD, Byeon JI, Dunn WA, Behrns KE, Leeuwenburgh C, Kim JS (2011). Autophagy suppresses age-dependent ischemia and reperfusion injury in livers of mice. Gastroenterology.

[29] Chang CP, Yang MC, Liu HS, Lin YS, Lei HY (2007). Concanavalin A induces autophagy in hepatoma cells and has a therapeutic effect in a murine in situ hepatoma model. Hepatology.

[30] Wu L, Feng Z, Cui S, Hou K, Tang L, Zhou J, Cai G, Xie Y, Hong Q, Fu B, Chen X (2013). Rapamycin upregulates autophagy by inhibiting the mTOR-ULK1 pathway, resulting in reduced podocyte injury. PloS one.

[31] Wu YT, Tan HL, Huang Q, Ong CN, Shen HM (2009). Activation of the PI3K-Akt-mTOR signaling pathway promotes necrotic cell death via suppression of autophagy. Autophagy.

[32] Blagosklonny MV (2008). Aging: ROS or TOR. Cell Cycle.

[33] Blagosklonny MV (2012). Rapalogs in cancer prevention: anti-aging or anticancer?. Cancer biology & therapy.

[34] Leontieva OV, Blagosklonny MV (2011). Yeast-like chronological senescence in mammalian cells: phenomenon, mechanism and pharmacological suppression. Aging.

[35] Wang C, Chen K, Xia Y, Dai W, Wang F, Shen M, Cheng P, Wang J, Lu J, Zhang Y, Yang J, Zhu R, Zhang H, Li J, Zheng Y, Zhou Y (2014). N-acetylcysteine attenuates ischemia-reperfusion-induced apoptosis and autophagy in mouse liver via regulation of the ROS/JNK/Bcl-2 pathway. PloS one.

[36] Comes F, Matrone A, Lastella P, Nico B, Susca FC, Bagnulo R, Ingravallo G, Modica S, Lo Sasso G, Moschetta A, Guanti G, Simone C (2007). A novel cell type-specific role of p38alpha in the control of autophagy and cell death in colorectal cancer cells. Cell death and differentiation.

[37] Zhao Y, Xue T, Yang X, Zhu H, Ding X, Lou L, Lu W, Yang B, He Q (2010). Autophagy plays an important role in sunitinib-mediated cell death in H9c2 cardiac muscle cells. Toxicology and applied pharmacology.

