# Supplementary material for: Autophagy protects against dasatinib-induced hepatotoxicity via p38 signaling
Source: Oncotarget. 2015 Jan 31;6(8):6203–17. doi: 10.18632/oncotarget.3357 (PMC4467432; doi:10.18632/oncotarget.3357)
Supplement: Supplementary file 1 [file oncotarget-06-6203-s001.pdf]

Autophagy protects against dasatinib-induced hepatotoxicity via p38 signaling

Supplementary Material

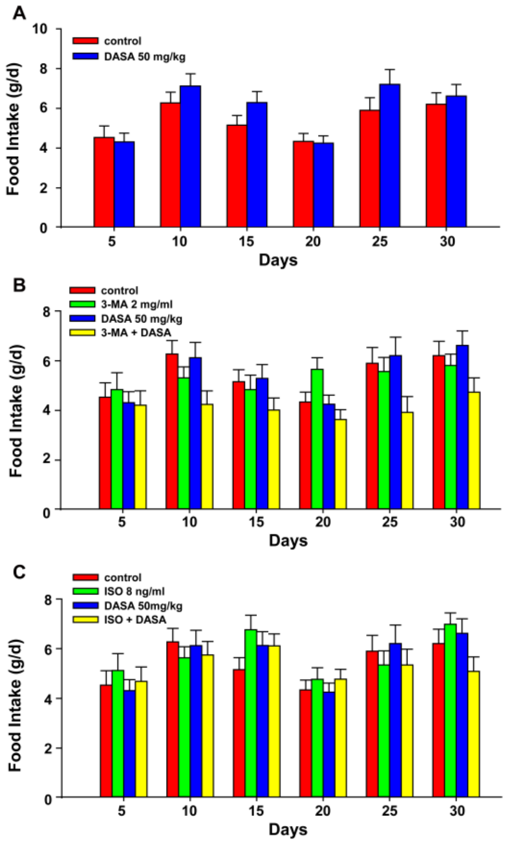

Figure S1: The food intakes of ICR mice were no change with dasatinib treatmment.

A. ICR mice were randomly divided into two groups (n=6) and were treated either with saline or dasatinib (50 mg/kg) for 30 days. Food intakes were calculated daily. B. ICR mice were randomly divided into 4 groups (n=6 mice) and treated with 3-MA (2 mg/kg), dasatinib (50 mg/kg) or both for 30 days. Food intakes were calculated daily. C.ICR mice were randomly divided into 4 groups (n=6 mice) and treated with ISO (8 ng/kg), dasatinib (50 mg/kg) or both for 30 days. Food intakes were calculated daily. Data are expressed as the mean ±SEM. Statistical analysis was performed by t-test where appropriate. DASA = dasatinib. ISO = isoproterenol hydrochloride.

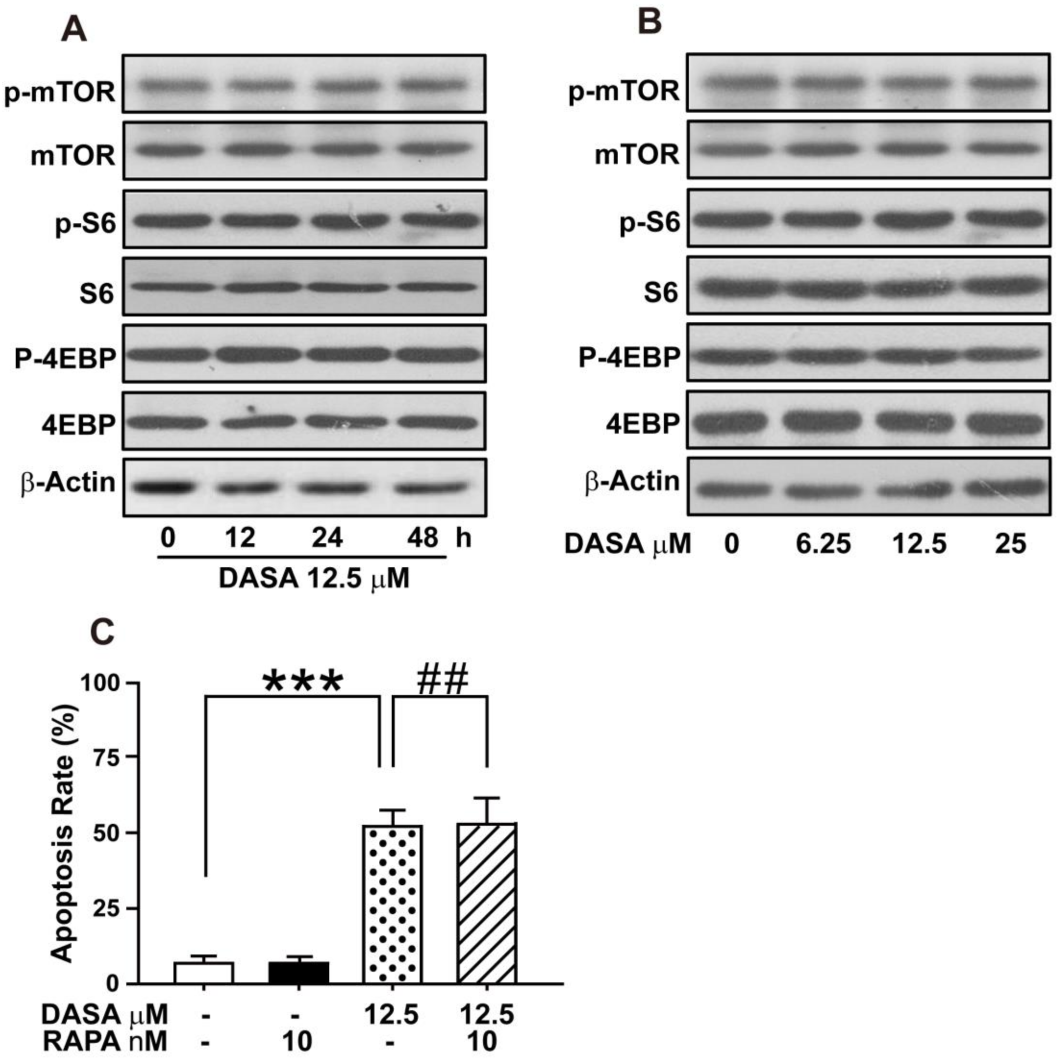

Figure S2: Dasatinib induces autophagy through a mTOR-independent pathway. (A-B) Hepatocytes were treated with dasatinib (12.5 μM) as indicated for 12, 24 or 48 hours or with different concentrations of DASA (0, 6.25, 12.5 and 25 μM) for 24 hours. Protein lysates of cells were subjected to western blot. C. Hepatocytes were treated with dasatinib (12.5 μM), RAPA (10 nM) or both for 24 hours. Apoptosis rate was determined by FACS.

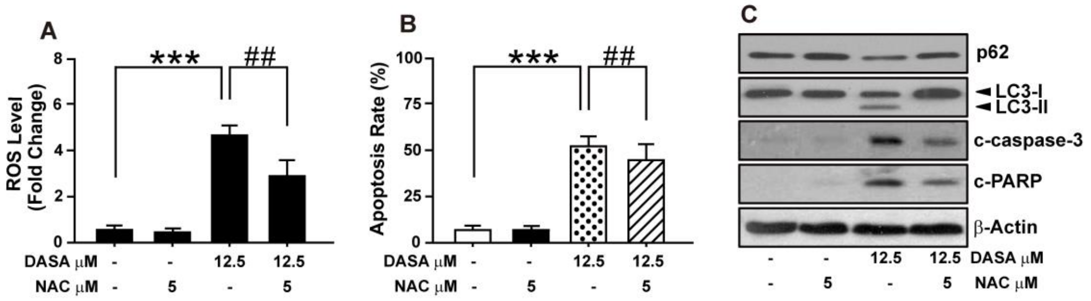

Figure S3: The antioxidant NAC attenuated dasatinib-induced hepatoxicity and autophagy. Hepatocytes were treated with dasatinib (12.5 μM), NAC (5 μM) or both for 24 hours. A. ROS level was detected using H2DCFDA and FACS. B. Apoptosis rate was determined by FACS. C. Protein lysates of cells were subjected to western blot.

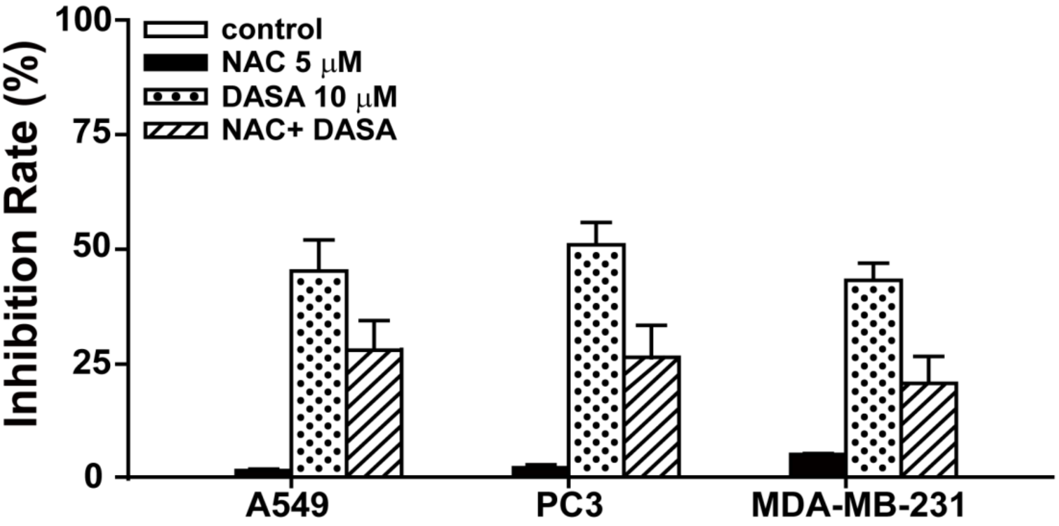

Figure S4: NAC antagonizes the anticancer activity of dasatinib. A. A549, PC3 and MDA-MB-231 cells were treated with NAC (5 μM), dasatinib (12.5 μM) or both for 24 hours. Apoptosis rate was determined by FACS following Annexin V and PI staining.
